# Local Induction Heating Capabilities of Zeolites Charged with Metal and Oxide MNPs for Application in HDPE Hydrocracking: A Proof of Concept

**DOI:** 10.3390/ma14041029

**Published:** 2021-02-22

**Authors:** Marta Muñoz, Irene Morales, Cátia S. Costa, Marta Multigner, Patricia de la Presa, Jose M. Alonso, João M. Silva, Maria do Rosário Ribeiro, Belén Torres, Joaquín Rams

**Affiliations:** 1 Department of Applied Mathematics, Materials Science and Engineering and Electronic Technology, Rey Juan Carlos University, 28933 Madrid, Spain; marta.multigner@urjc.es (M.M.); belen.torres@urjc.es (B.T.); joaquin.rams@urjc.es (J.R.); 2 Institute of Applied Magnetism, UCM-ADFI-CSIC, 28230 Las Rozas, Spain; irenemorales@ucm.es (I.M.); pmpresa@ucm.es (P.d.l.P.); jm.a.r.0@csic.es (J.M.A.); 3 Centro de Química Estrutural (CQE), Instituto Superior Técnico, Universidade de Lisboa, 1049-001 Lisbon, Portugal; catia.s.costa@tecnico.ulisboa.pt (C.S.C.); jmsilva@deq.isel.ipl.pt (J.M.S.); rosario@tecnico.ulisboa.pt (M.d.R.R.); 4 Department of Materials Physics, Complutense University of Madrid, 28040 Madrid, Spain; 5 Material Science Institute of Madrid, CSIC, 28049 Madrid, Spain; 6 Instituto Superior de Engenharia de Lisboa (ISEL), Instituto Politécnico de Lisboa, 1959-007 Lisboa, Portugal

**Keywords:** Zeolite, plastic waste, hydrocracking, hyperthermia, magnetic nanoparticles (MNPs), electromagnetic fields

## Abstract

Zeolites are widely used in high-temperature oil refining processes such as fluid catalytic cracking (FCC), hydrocracking, and aromatization. Significant energy cost are associated with these processes due to the high temperatures required. The induction heating promoted by magnetic nanoparticles (MNPs) under radio frequency fields could contribute to solving this problem by providing a supplementary amount of heat in a nano-localized way, just at the active centre site where the catalytic process takes place. In this study, the potential of such a complementary route to reducing energetic requirements is evaluated. The catalytic cracking reaction under a hydrogen atmosphere (hydrocracking) applied to the conversion of plastics was taken as an application example. Thus, a commercial zeolite catalyst (H-USY) was impregnated with three different magnetic nanoparticles: nickel (Ni), cobalt (Co), maghemite (γ-Fe_2_O_3_), and their combinations and subjected to electromagnetic fields. Temperature increases of approximately 80 °C were measured for H-USY zeolite impregnated with γ-Fe_2_O_3_ and Ni-γ-Fe_2_O_3_ due to the heat released under the radio frequency fields. The potential of the resulting MNPs derived catalyst for HDPE (high-density polyethylene) conversion was also evaluated by thermogravimetric analysis (TGA) under hydrogen atmosphere. This study is a proof of concept to show that induction heating could be used in combination with traditional resistive heating as an additional energy supplier, thereby providing an interesting alternative in line with a greener technology.

## 1. Introduction

Plastic production increased by more than 20-fold from 1964 to 2018 with an annual output of 359 Mt, [1]. This value is expected to reach 18,000 Mt in 2050 [2]. Despite the contribution of plastics to the economic evolution of the world, their current production and use patterns are leading to serious environmental problems [3].

Hydrocracking technology has been recognized as a very promising solution for managing plastic waste since it allows plastic feedstock to be converted into valuable products, thus removing heteroatoms that may exist in waste plastic, reducing the amount of olefins and aromatics in the final products, and reducing the coke precursors responsible for the catalyst deactivation in one single process [4,5]. Typically, the hydrocracking catalysts are bifunctional, comprising an acidic function responsible for the cracking and isomerization reactions, and metal centers where hydrogenation-dehydrogenation reactions take place [6]. The acidic function is generally given by amorphous oxides (silica-alumina), zeolites, strong solid acids (sulfated zirconia) or a combination of these materials. Nevertheless, zeolites are by far the most applied materials because they have unique properties: high thermal stability, high strength and number of acidic sites, high external surface area, and an unequalled pore channel system, all of which make them suitable catalysts for plastic conversion [7,8,9,10,11]. In turn, the metallic function is usually represented by a noble metal (palladium or platinum), a non-noble metal of group VI-A (molybdenum or tungsten) or group VIII-A (cobalt or nickel), according to the periodic table [5].

The conversion of a single type or a mixture of plastics over bifunctional catalysts [7,8,12,13,14,15,16,17,18] by hydrocracking has been reported by several authors. Their findings show that the presence of a catalyst has a beneficial effect in the reaction, decreasing the temperature and the time required to achieve high conversions and allowing enhancing the quality of the gas and liquid products. Unfortunately, the energy requirements associated with this process are still high.

In order to address this problem, magnetic nanoparticles (MNPs) under radio-frequency fields are proposed for use as nanoheaters. MNPs offer a simple way of achieving high, fast and nano-localized temperatures [19] that are able to participate in these processes as an additional energy supplier, thus decreasing significantly the conventional heating requirements.

Numerous studies in the literature discuss the use of high-frequency fields for heating MNPs in different areas as medical applications (e.g., cancer therapies) [20,21], engineering and technological applications like water electrolysis reactions [19], CO_2_ hydrogenation and methanation [22,23], and functions related to polymeric materials like induction heating polymerization of molecularly imprinted polymers [24]. Electromagnetic fields in combination with MNPs are also used as heat sources for the suitable processing of thermoplastic and thermoset polymers [25,26], rapid curing of epoxy resins [27], and many other applications where high temperatures are required.

Some of these investigations [19,23,28] refer to a wide number of MNP compositions for induction-heating applications. In particular, iron carbide nanoparticles display an exceptionally high heating power under alternating electromagnetic fields [28]. Despite the high heating efficiency of iron carbide MNPs, one of their main limitations lies in the complexity of their synthesis, which makes it difficult to scale up. However, maghemite (γ-Fe_2_O_3_) nanoparticles combine in an easy and economical synthetic procedure with high magnetic moments, and it is well established that they are able to generate thermal energy under alternating magnetic fields. Nickel- and cobalt-reduced metal nanoparticles also show suitable magnetic properties (Table 1), but little information is available in the literature regarding their efficiency as magnetic heating agents. Due to the difficulty of stabilizing metallic MNPs, most of the literature refers to their oxides, alloys, or functionalized forms [29,30,31,32,33,34].

In order to determine the potential application of γ-Fe_2_O_3_ nanoparticle-impregnated H-USY zeolites for catalytic reactions promoted by acid catalyst under electromagnetic fields, their heating efficiency was previously investigated [36].

In this work, we extended our previous studies to other MNPs, single or combined with γ-Fe_2_O_3_, with the aim of introducing new metal centers that may combine catalytic and ferromagnetic properties for potential applications in the catalytic conversion of high-density polyethylene (HDPE) under hydrogen atmosphere (hydrocracking). Since Ni and Co metallic nanoparticles are commonly used in hydrocracking catalysts, they were selected for this study. It is also worth mentioning that, due to the reductive conditions used in hydrocracking, the reduction of γ-Fe_2_O_3_ to metallic iron would be expected to lead to a greater heating efficiency.

Therefore, H-USY zeolite was impregnated with three different MNPs: nickel, cobalt, maghemite and their combinations and then subjected to electromagnetic fields in order to determine the efficiency of the heating induction, the possibility of synergic effects, and the potential of the resulting MNP-derived catalysts for HDPE conversion under hydrogen atmosphere. Induction heating assays were used to compare the heating efficiencies of the different MNP-impregnated zeolites. Experimental parameters involved in the heating capability of these systems such as magnetic field, amplitude, and frequency were systematically investigated. Moreover, preliminary evaluation of the catalytic performance of the MNP-based zeolites was also carried out through thermogravimetric analysis.

## 2. Materials and Methods

### 2.1. Materials

The commercial H-USY zeolite with a Si/Al ratio of 40 (CBV780) was supplied by Zeolyst in a powder form.

Nickel nitrate hexahydrate (Ni(NO_3_)_2_·6H_2_O (Merck, Darmstadt, Germany, >99%) and cobalt (II) acetate (Sigma Aldrich, St. Louis, MO, USA, >99%) were used as precursor salts for Ni and Co, respectively.

Commercial high-density polyethylene in powder form and with no additives (HDPE, MW = 155,000 g/mol; D = 5.4; d = 0.95 g/cm^3^ and Tm = 140 °C) was kindly supplied by Repsol (Sines, Portugal). 

### 2.2. MNPs Synthesis

The γ-Fe_2_O_3_ MNPs were prepared using the Massart modified coprecipitation method, which allows a large quantity of material to be obtained in a cheap way [37]. Briefly, 12 nm γ-Fe_2_O_3_ nanoparticles were prepared by mixing 0.09 mol of iron (III) chloride 6-hydrate and 0.054 mol of iron (II) chloride 4-hydrate in a total volume of 488 mL of distilled water. This solution was added slowly (0.2 mL/s) into 75 mL of a base solution (NH_4_OH 25%) under constant magnetic stirring. The mixture was heated to 90 °C and left at this temperature for 1 h. Next, the black product (Fe_3_O_4_) was washed several times, by magnetic decantation with distilled water to get rid of the supernatant.

For the oxidation of Fe_3_O_4_ into γ-Fe_2_O_3_, 300 mL of HNO_3_ (2M) was added to the washed nanoparticles and magnetically stirred for 15 min. Then, the supernatant was removed and a 75 mL aqueous solution of iron (II) nitrate 9-hydrate (1M) and 130 mL of distilled water were added. The mixture was heated to a boiling temperature for 30 min. The supernatant was again removed by magnetic decantation and 300 mL of HNO_3_ 2M was added and mixed for 15 min. Finally, the obtained γ-Fe_2_O_3_ nanoparticles were washed with acetone three times. The acetone was evaporated in a rotary evaporator and the nanoparticles were redispersed in distilled water. This second step allows not only the oxidizing of the nanoparticles but also the dissolving of the smallest nanoparticles and the recrystallizing of the larger ones thereby obtaining a narrower distribution.

### 2.3. MNP-Based Catalysts Preparation

H-USY zeolites were impregnated and co-impregnated with MNPs (Ni, Co, γ-Fe_2_O_3_, Ni–γ-Fe_2_O_3,_ and Co–γ-Fe_2_O_3_), using the incipient wetness impregnation method in order to obtain the corresponding MNP-based catalysts.

For mono-, Ni-, Co- and γ-Fe2O3-impregnated catalysts, a 1.7 mL aqueous solution of Ni(NO_3_)_2_·6H_2_O (C = 155 g/L), (CH_3_CO_2_)_2_Co (C = 132 g/L) and γ-Fe2O3 (C_Fe_ = 72.2 g/L) was added, respectively, drop by drop to the H-USY(40) zeolite. The samples were air-dried at 80 °C for 24 h, and posteriorly calcined under airflow (4 L×h^−1^×g^−1^) at 500 °C. The catalysts preactivation was performed in a glass reactor under hydrogen flow (4 L×h^−1^×g^−1^) at 500 °C for 2 h. These last two steps were not performed for γ-Fe_2_O_3_ /H-USY(40) in order to avoid the transformation of maghemite into hematite.

For the co-impregnated zeolites (Ni–γ-Fe_2_O_3_ and Co–γ-Fe_2_O_3_), the Ni and Co were impregnated first and then the γ-Fe_2_O_3_. In this case, 1.7 mL of Ni(NO_3_)_2_·6H_2_O (C = 75.5 g/L) or (CH_3_CO_2_)_2_Co (C = 64.4 g/L) aqueous solution was added to the H-USY(40) zeolite. Then, the impregnated samples were air dried, calcined and pre-activated under the above-mentioned conditions. The mono-impregnated Ni/H-USY(40) and Co/H-USY(40) were further co-impregnated through the addition of a 1.7 mL aqueous solution of γ-Fe_2_O_3_ (C_Fe_ = 35.1 g/L) and dried at 80 °C for 24 h.

All materials were prepared with a total metal content of 5 wt.%.

### 2.4. HDPE Films Preparation

The HDPE was mechanically mixed with MNP-based catalysts in a polymer-to-catalyst mass ratio of 7.5/2.5. The mixture was processed into films by compression molding in a Specac hydraulic press (Specac, UK) at 140 °C for 2 min without pressure and then for 3 min at 3 ton.

### 2.5. MNP-Based Cataysts Characterization

Textural properties of the prepared catalysts were evaluated from nitrogen adsorption–desorption isotherms obtained at −196 °C using an Autosorb IQ apparatus from Quantachrome (Boynton Beach, FL, USA) Prior to adsorption, the samples were heated under vacuum at 90 °C for 1 h and then at 350 °C for 5 h. The t-plot method was used for the determination of the external surface area (S_ext_) and for the microporous volume (V_micro_). The total pore volume (V_total_) was calculated from the volume of adsorbed N_2_, at a relative pressure (P/P_o_) of 0.95. The mesoporous volume (V_meso_) was calculated by the difference between V_total_ and V_micro_.

A transmission electron microscope (TEM) JEOL-JEM 2100F (JEOL, Tokyo, Japan) operating at 200 keV, was used to study the morphology, particle size, and distribution of the nanoparticles in the H-USY(40) zeolite.

The Powder X-ray diffraction (PXRD) analysis was performed in a Bruker AXS Advance D8 diffractometer (Billerica, MA, USA) equipped with a 1D detector (SSD 160) and operating at 30 mA and 40 kV. A Ni filter, and a radiation source of CuKα (λ = 1.5406 nm) was used and a scanning range from 5 to 80° (2Ɵ), with a step of 0.03°/2 s was defined.

### 2.6. Thermogravimetric Experiments

The HDPE degradation experiments over distinct catalytic systems were carried out in a Setaram TGA 92-16.18 equipment under H_2_ atmosphere a flow rate of 30 mL/min. The temperature was varied from 20 to 700 °C, at a rate of 10 °C/min. A nitrogen purge was performed before each experiment, to avoid the presence of oxygen.

### 2.7. Induction Heating Assays

Heating properties were measured with a commercial Magnetherm 1.5 (Nanotherics) device (Nanotherics, Warrington, UK). The system is composed of a 17-turn coil with 5 different capacitors, which allows working with different resonance frequencies and studying the effect of the magnetic field and the frequency in the heating release of the nanoparticles. The coil temperature was maintained at 16 °C with a LAUDA Alpha RA12 peristaltic device (Lauda Dr. R. Wobser GMBH & Co. KG, Lauda-Königshofen, Germany). The temperature increase of the nanoparticle-impregnated zeolite was measured with a thermographic camera FLIR E53 (FLIR^®^ Systems, Inc., Wilsonville, OR, USA), field of vision 24° × 18° Lens, (240 × 180 pixels resolution) and registered in the computer. Once the thermal stability was reached and before turning the magnetic field on, temperature was registered for 30 s to obtain the baseline, afterwards the field was switched on. The slope of the heating curve was calculated for the first 30–50 s after the field was turned on at the maximum field for each frequency.

## 3. Results and Discussion

### 3.1. Catalyst Characterization

**Figure 1:**
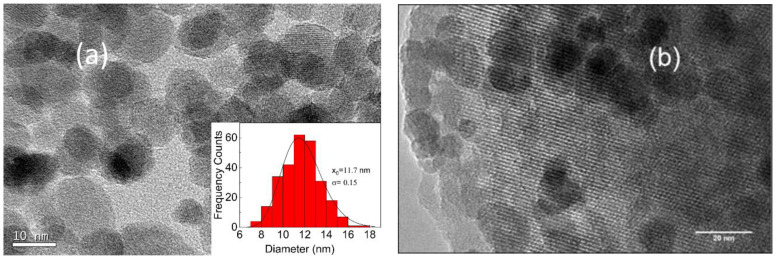
TEM of (**a**) γ-Fe_2_O_3_ nanoparticles with the corresponding histogram and (**b**) γ-Fe_2_O_3_/H-USY(40).

The incorporation of a Ni metal source with a zeolite material is a very common procedure in the literature, resulting generally in well-dispersed Ni particles in the supports [38]. Nevertheless, the incorporation of **γ**-Fe_2_O_3_ in zeolite is a less studied topic [36]. Figure 1a displays TEM images of the nanoparticles along with their size distribution fitted to a lognormal distribution; the inset shows the histogram of around 200 nanoparticles exhibiting a mean particle size of d = 11.7 nm and a polydispersity degree (standard deviation/mean size) σ = 0.2, as expected [37]. Figure 1b shows the TEM images of **γ**-Fe_2_O_3_/H-USY. It can be seen that **γ**-Fe_2_O_3_ nanoparticles are well dispersed in the H-USY(40) zeolite. In addition, the pore structure of this zeolite is clearly visible.

**Figure 2:**
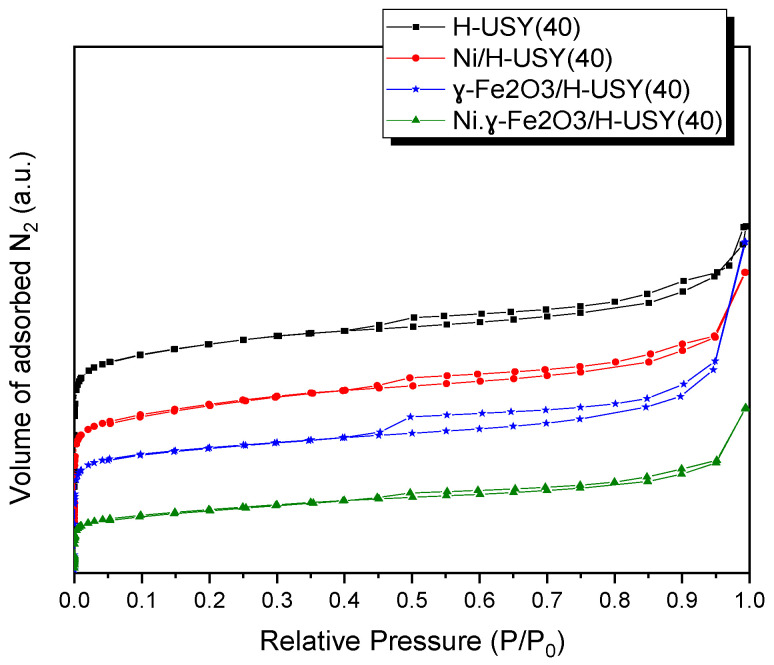
N_2_ sorption isotherms of parent and MNP-based H-USY(40) zeolites.

The textural properties of distinct MNP-based catalysts were evaluated from the N_2_ sorption measurements (Figure 2). According to the IUPAC classification [39], the H-USY(40) zeolite exhibits a combination of a type I and type IV isotherms typical of microporous and mesoporous materials, respectively [40]. According to the literature [41], the appearance of some mesoporosity in H-USY zeolites, which is typical of microporous materials, is related to the post-modification dealumination treatments used for this family of zeolites with distinct Si/Al ratios.

Upon the MNP-impregnation process, nonsignificant modifications were observed on the isotherms shape, revealing the preservation of the porous structure. Nevertheless, the external surface area and the microporous and mesoporous volume are reduced for the H-USY(40) zeolites impregnated with γ-Fe_2_O_3_ (Table 2), indicating the deposition of MNPs either on the catalyst external surface area or in the of the zeolite pores, corroborating the TEM results [42,43]. For the Ni impregnated catalysts the variations on the textural properties are less pronounced.

**Figure 3:**
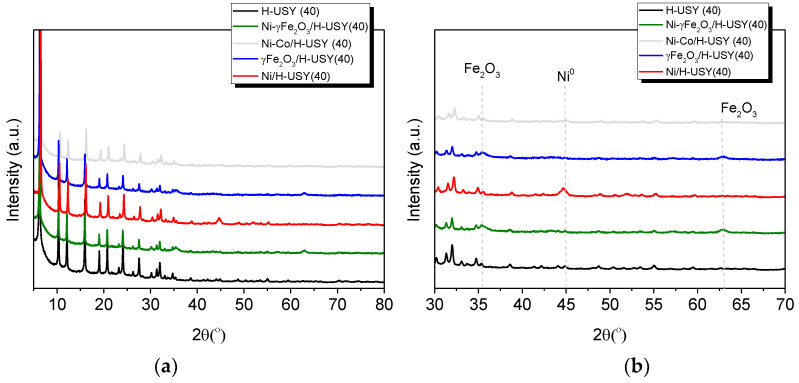
(**a**) XRD patterns of parent and MNPs impregnated H-USY(40) zeolites; (**b**) Magnification.

The PXRD results of parent and MNP- (Ni, γ-Fe_2_O_3_ and Ni–γ-Fe_2_O_3_) based H-USY(40) impregnated zeolites are displayed in Figure 3. The data revealed similar PXRD patterns for parent and MNP-impregnated catalysts with peaks at 2θ = 6.2° (110), 12.1° (311), 15.9° (400), 18.9° (333), 20.7° (440), 27.0° (642) and 27.5° (731), typical of faujasite (FAU) structure. This indicates that the structure was not modified after the impregnation procedure thus, corroborating the N_2_ sorption data. Additional reflections, characteristic of metallic nickel (Ni) and γ-Fe_2_O_3_ are detected respectively, at 2θ = 45° (111) and 2θ = 35.6° (311) and 62.8° (440). In the case of Ni–γ-Fe_2_O_3_/H-USY(40) sample, no characteristic peaks belonging to Ni^0^ are detected, probably because a smaller amount of Ni was used in this catalyst (2.5 wt.%).

### 3.2. Degradation Experiments

**Figure 4:**
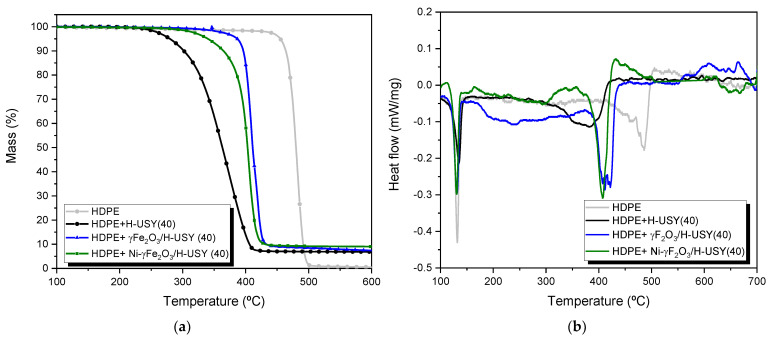
TGA (**a**) and heat flows (**b**) profiles for the catalytic degradation of HDPE under H_2_ atmosphere over parent and MNPs impregnated H-USY(40) zeolites.

TGA is widely used as a tool to assess the potential of distinct catalytic systems to promote the degradation of polymers, especially concerning energy requirements [44,45]. In the present study, the effectiveness of the MNP-catalytic systems for HDPE degradation under reductive conditions was evaluated using TGA. The mass loss and heat-flow profiles are displayed in Figure 4, and the temperatures at which mass loss is 5, 50 and 95% (T_5%_, T_50%_ and T_95%_) are summarized in Table 3.

The data showed that thermal degradation of HDPE occurred in a single mass-loss step, exhibiting a degradation range between 433 and 488 °C, and corroborates the high-energy consumption associated with HDPE conversion. The addition of a catalyst facilitates HDPE degradation, shifting the degradation profiles to lower temperature values. The highest shift was observed for H-USY(40), which allowed for a diminishment of 184 °C on T_5%_ when compared to the thermal run.

The introduction of γ-Fe_2_O_3_ in the H-USY(40) zeolite resulted in an increase of T_5%_, T_50%_ and T_95%_ relative to the parent zeolite, which may be attributed to a decrease in the accessibility of HDPE macromolecules to the porous zeolite framework. N_2_ sorption data corroborated this assumption. As already mentioned, a reduction in the textural properties (S_ext_, V_micro_, V_meso_) was observed, indicating the depositing of γ-Fe_2_O_3_ on the H-USY surface and on the porous structure, thus hindering the access of the polymer to the active centers. Although γ-Fe_2_O_3_ is not a suitable metallic center for the hydrocracking process, as it does not improve the degradation process, it is essential for the reduction of energy consumption through a magnetic-induced field.

In turn, the association of Ni to the previous γ-Fe_2_O_3_/H-USY(40) system leads to a decrease of T_5%_, and to a reduction in the energy requirements of the process. The beneficial role of Ni on hydrocracking reactions was already reported in a previous publication [38]. In contrast to γ-Fe_2_O_3_, Ni exhibits a good dehydrogenation–hydrogenation ability, which is essential for hydrocracking reactions. Despite the positive effect of Ni, T_5%_ still remains higher than the value observed for the parent zeolite, and further research will be necessary to avoid the loss of the active center’s accessibility and to improve the catalytic behavior of MNP-impregnated catalysts.

The heat flow profiles, showed in Figure 4b reveal the presence of two distinct endothermic peaks. The first, around 140 °C, is due to the melting of the polymer. The second one corresponds to the degradation of HDPE and, therefore, is accompanied by a mass loss. Unlike the first peak, which appears at the same temperature, the position of the second peak is strongly influenced by the presence or absence of a catalyst and also by the catalyst nature. In this case, the most promising catalytic systems for converting HDPE exhibit the degradation peak at lower temperatures.

Since hydrocracking reactions are performed in a reductive atmosphere, it was also found important to evaluate the stability of mono- and co-impregnated zeolites under these conditions. In a previous study [36] on the stability of parent γ-Fe_2_O_3_ MNPs and γ-Fe_2_O_3_ MNPs impregnated in H-USY(40) zeolite under hydrogen atmosphere, it was found that the γ-Fe_2_O_3_ nanoparticles exhibited two distinct mass losses: one at 344–444 °C and the other at 444–634 °C, corresponding respectively, to the transformation of γ-Fe_2_O_3_ to Fe_3_O_4_ and its subsequent reduction to metallic Fe. In turn, when γ-Fe_2_O_3_ was impregnated in H-USY(40) zeolite, a continuous reduction pattern was observed. A similar behavior can be observed for the co-impregnated Ni–γ-Fe_2_O_3_/H-USY(40) zeolite displayed in Figure S1 of SUP INF. As Ni nanoparticles, both in mono- and co-impregnated H-USY(40) zeolite, were already in a reduced form, no further changes were expected to occur under hydrogen atmosphere.

### 3.3. Induction Heating Assays

Taking into account the promising exploratory results obtained for HDPE degradation, the previous MNP-impregnated H-USY systems were subjected to induction heating experiments, and the heating rate of the MNPs supported on the zeolite under radio-frequency fields was evaluated. These tests were also extended to Co and Ni–Co-impregnated H-USY(40) zeolites in order to evaluate possible synergetic effects in the magnetic properties. Five different combinations of three nanoparticles with high values of magnetic susceptibility (γ-Fe_2_O_3_, Ni and Co) were analyzed.

The experiments were performed in an air atmosphere, but more positive results are expected under hydrogen atmosphere (used in the hydrocracking process), where maghemite is reduced to metallic iron with a higher saturation magnetization (Table 1). Consequently, higher temperatures will be reached.

#### 3.3.1. Induction Heating on Impregnated H-USY(40)

H-USY(40) zeolites impregnated with MNPs (powder) were manufactured in pellets with a hydraulic press. The concentration of the MNPs with respect to the mass of zeolite is 5% for most samples. In the case of Ni–γ-Fe_2_O_3_ and Ni–Co mixtures, 2.5% of each metal was used to reach the total concentration (5%) required.

According to previous work [46], the heating efficiency was evaluated by means of the Specific Absorption Rate (SAR) as

(1) $$SAR=\frac{Cz}{\left[Fe\right]}\frac{∆T}{∆t}$$

where *Cz* is the heat capacity of zeolite (unknown), [*Fe*] is the iron mass concentration, *∆T* is the temperature increment, *∆t* is the time increase and *∆T/∆t* is the initial slope of the heating curve.

SAR calculation requires the knowledge of the heating capacity of the zeolite (Equation (1)), which is unknown in this case. Assuming that the heating capacity (*Cz*) of the H-USY(40) zeolite is the same for all the samples, the initial slope, *∆T*/*∆t*, of the heating curve was used to calculate the heating rate per Fe unit mass of each sample, *∆T*/*∆t*/[Fe].

The heating curves for all the samples at three different frequencies 112, 627 and 990 kHz (low, medium and high) are displayed in Figure S2 in SUP INF. The samples showing a significant heating rate are those impregnated with γ-Fe_2_O_3_ and Ni–γ-Fe_2_O_3_ whereas those, containing Ni and Ni–Co exhibited a negligible temperature increase, probably due to the oxidation suffered by Ni and Co metals under air atmosphere.

In Table 4 it is shown the heating rate/unit mass obtained for H-USY(40) impregnated with γ-Fe_2_O_3_ and Ni–γ-Fe_2_O_3_ at 10 different frequencies. Based on these results it was decided to perform induction heating tests at the same frequency: 526 kHz and 100 Oe for all samples. Although the *∆T*/*∆t*)/[*Fe*] value obtained for Ni-γ-Fe_2_O_3_ system was slightly higher at 112 kHz than at 526 kHz the difference is rather small. The data in this Table also suggest a positive synergistic effect between Ni and γ-Fe_2_O_3_ since the heating rate per Fe mass increases 50% under the presence of Ni.

**Figure 5:**
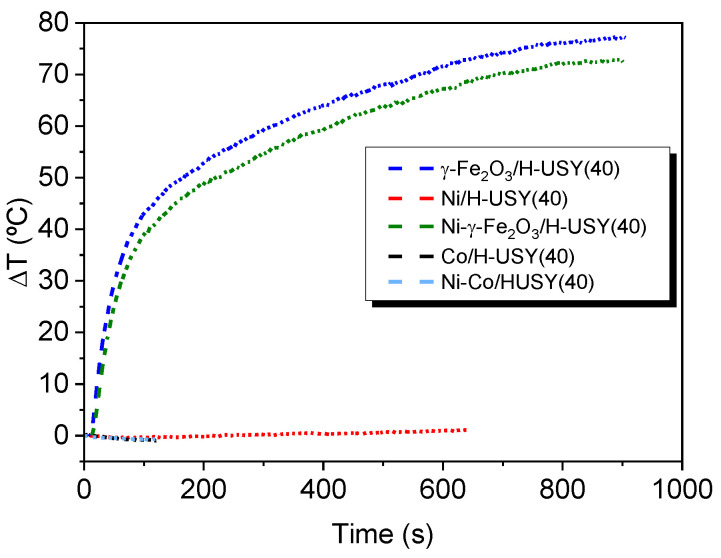
Induction heating curves (526 kHz) of H-USY(40) impregnated with γ-Fe_2_O_3_ (Dark blue), Ni–γ-Fe_2_O_3_ (Green), Ni (Red), Ni–Co mixture (Light blue) and Co (Black).

Figure 5 shows the induction heating curves for zeolite H-USY(40) impregnated with γ-Fe_2_O_3_ (blue), Ni (red) and the Ni–γ-Fe_2_O_3_ (green) MNPs at 5%, at 526 kHz and 100 Oe. A temperature increase about 75 °C in 800 s is observed for the γ-Fe_2_O_3_-impregnated system and 70 °C for the Ni–γ-Fe_2_O_3_ one. The results obtained for the γ-Fe_2_O_3_-impregnated zeolite were quite similar to those referred to in a previous publication [36].

Even though the thermographic camera detected a global temperature increase of approximately 75 °C, it is probable that the local temperature reached at the MNPs surface was much higher because they emitted heat to the rest of the sample and the polymer used had a low thermal conductivity.

#### 3.3.2. Heating Induction on HDPE and Reinforced Catalysts Films

Additional induction-heating experiments were performed over HDPE–zeolite–MNP films in order to determine the respective heating rate under radio-frequency fields. Films containing 25 wt.% of a catalyst were subjected to a systematic frequency sweep to find the maximum degree of induction heating on each film (see supporting information Figure S2a–c). Similarly to the previous results, only the films containing H-USY impregnated with γ-Fe_2_O_3_ and Ni–γ-Fe_2_O_3_ at 526 kHz showed a significant temperature increase. 

**Figure 6:**
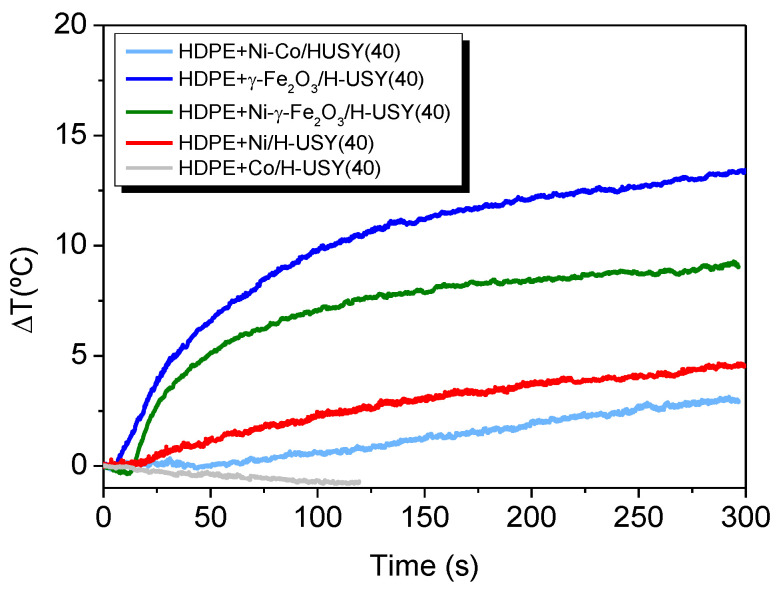
Temperature increment curves (526 kHz) for HDPE films containing H-USY(40) impregnated with: γ-Fe_2_O_3_ (Dark blue), Ni–γ-Fe_2_O_3_ (Green), Ni (Red), Ni–Co mixture (Light Blue) and Co (Grey) over time.

Figure 6 displays the temperature variation as a function of time for the HDPE–zeolite–MNPs films at 526 kHz: γ-Fe_2_O_3_ is shown in red and Ni–γ-Fe_2_O_3_ in blue. A smaller temperature increment was observed for HDPE films compared to the parent MNP-impregnated zeolites. This was due to a decrease in the concentration of nanoparticles in the HDPE films by a factor of four.

Table 5 displays the variation of the heating rate/unit mass with frequency for the HDPE films. The optimal frequency at which maximum degree of induction heating occurs is the same as observed for the impregnated zeolite systems in the previous section.

In addition, as shown in Table 4 and Table 5, the temperature increase per mass concentration (*∆T*/*∆t*)/[*Fe*] for γ-Fe_2_O_3_- and Ni–γ-Fe_2_O_3_-impregnated H-USY(40) zeolites and for the corresponding HDPE–zeolite–MNP films is very similar and shows its maximum at 526 kHz. These results suggest that the film preparation does not affect the heating efficiency of nanoparticles.

The Ni–γ-Fe_2_O_3_ and γ-Fe_2_O_3_ based systems show a considerable temperature increase per Fe mass of 29.71 and 14.86 K/s respectively. Since the amount of magnetic Fe cations in Ni-γ-Fe_2_O_3_ is the half of γ-Fe_2_O_3_, and Ni nanoparticles do not show any temperature increase, which suggests an extra contribution to the heating, thus corroborating the synergistic effect of Ni and γ-Fe_2_O_3_ as proposed in the previous section.

## 4. Conclusions

In this work, we investigated the effect of adding several types of MNPs (Ni, Co and γ-Fe_2_O_3_) to a zeolite catalyst to determine the local heating ability of these MNPs and the degradation of HDPE under hydrogen atmosphere promoted by these MNP-derived catalysts.

The combination of catalytic and magnetic properties in these new materials offered one interesting advantage: the instantaneous transfer of heat directly to the active sites of the catalyst so that the heat source––the MNPs subjected to alternating magnetic fields—came from inside the zeolite, which should minimize the energy loss of conventional heating systems with external energy suppliers.

The results shown here demonstrated that under an electromagnetic field (work frequency of 526 kHz and air atmosphere) the combination of Ni and γ-Fe_2_O_3_ with the H-USY(40) zeolite permitted the rapid increase of the local temperature up to 80 °C. Moreover, the effectiveness of the heating induction process was expected to be improved under the hydrogen atmosphere used in the hydrocracking process because of the reduction of maghemite to metallic iron with a higher saturation magnetization.

This preliminary investigation can be taken as a proof of concept and a first indication of the potential of MNP-derived zeolite catalysts for use as a complimentary heating source for the catalytic conversion of HDPE under H_2_ atmosphere. The approach developed here may provide an interesting auxiliary route to reducing the energetic requirements of catalytic processes and the environmental impact of plastic waste, which is in line with EU policy.

Further research, both regarding the development of more efficient magnetic nanoparticles and more effective catalysts, is necessary for a full assessment of MNP-derived catalyst abilities. The application of this concept to other high energy-consuming catalytic processes is another possibility.

## 5. Patents

This section is not mandatory but may be added if there are patents resulting from the work reported in this manuscript.

## Figures and Tables

**Table 1:** Saturation magnetization (Ms) and Curie Temperature for various materials [35].

Material	Ms (emu/g)		Curie Temperature (°C)
	0 K	293 K	
Fe	221.9	218.0	770
Co	162.5	161	1131
Ni	57.5	54.4	358
γ-Fe_2_O_3_	83.5	76	Unstable
Fe_3_O_4_	98.0	92	585

**Table 2:** Textural properties determined by N_2_ adsorption/desorption for parent H-USY(40) and MNP-based zeolites.

Catalyst Type	S_ext_ (m^2^/g)	V_micro_ (cm^3^/g)	V_meso_ (cm^3^/g)	V_total_ (cm^3^/g)
H-USY(40)	251	0.210	0.250	0.460
Ni/H-USY(40)	255	0.147	0.250	0.397
γ-Fe_2_O_3_/H-USY(40)	168	0.185	0.235	0.420
Ni–γ-Fe_2_O_3_/H-USY(40)	155	0.063	0.165	0.228

**Table 3:** Comparison of T5%, T50% and T95% for HDPE degradation on H-USY(15) catalyst at 15%.

Sample	T_5%_ (°C)	T_50%_ (°C)	T_95%_ (°C)
HDPE	433	478	488
HDPE + H-USY(40)	270	357	399
HDPE+ γ-Fe_2_O_3_/H-USY(40)	384	409	425
HDPE+ Ni/γ-Fe_2_O_3_/H-USY(40)	335	401	418

**Table 4:** Slope, (*∆T*/*∆t*)/Fe, of the induction heating curve for H-USY(40) impregnated with γ-Fe_2_O_3_ and Ni–γ-Fe_2_O_3_ zeolites.

Frequency (kHz)	Field (Oe)	γ-Fe_2_O_3_ (*∆T*/*∆t*)/[*Fe*] (K/s)	Ni–γ-Fe_2_O_3_ (*∆T*/*∆t*)/[*Fe*] (K/s)
112	172	14.57	38.29
165	133	8.57	22.86
177	124	12.57	21.14
263	73	18.29	28.00
331	60	22.29	34.86
468	73	11.71	20.00
526	94	23.43	35.43
625	50	6.00	12.00
740	35	6.57	9.14
990	32	4.00	6.86

**Table 5:** Slope (*∆T*/*∆t*)/Fe of the induction heating curve for HDPE films containing H-USY(40) impregnated with γ-Fe_2_O_3_ and Ni–γ-Fe_2_O_3._

Frequency (kHz)	Field (Oe)	γ-Fe_2_O_3_ (*∆T*/*∆t*)/[*Fe*] (K/s)	Ni-γ-Fe_2_O_3_ (*∆T*/*∆t*)/[*Fe*] (K/s)
112	172	12.57	16.00
165	133	5.71	9.14
177	124	8.00	9.14
263	73	10.29	18.29
331	60	13.71	20.57
468	73	5.71	9.14
526	94	14.86	29.71
625	50	2.29	2.29
740	35	1.14	4.57
990	32	1.14	0.00

## Data Availability

Data available in a publicly accessible repository.

## Supplementary Materials

The following are available online at https://www.mdpi.com/1996-1944/14/4/1029/s1. Figure S1: TGA of Ni and Ni–γ-Fe2O3 impregnated H-USY(40) zeolites, under hydrogen atmosphere; Figure S2a: Heating curves of all the samples: films with HDPE (continuous lines) and pellets (dashed lines) at 112 kHz; Figure S2b: Heating curves of all the samples (films and pellets) at 627 kHz; Figure S2c: Heating curves of all the samples (films and pellets) at 990 kHz.

## Abbreviations

Cz	Heat capacity
d	Diameter
D	Molecular weight dispersion
FTIR	Fourier transform infrared spectroscopy
FCC	Fluid catalytic cracking
HDPE	High density polyethylene
IMA	Institute of Magnetism Applied
IST	Institute Superior Technique
MNC	Magnetic nanoparticle concentration
MNP	Magnetic nanoparticle
Ms	Saturation magnetization
MW	Molecular weight
MWD	Molecular weight distribution
SAR	Specific absorption rate
SEM	Scanning electron microscopy
Sext	External surface area
TEM	Transmission electron microscopy
TGA	Thermogravimetric analysis
Tm	Melting temperature
UCM	University Complutense of Madrid
Vmeso	Mesoporous volume
Vmicro	Microporous volume
Vtotal	Total pore volume
W	Weight
PXRD	Powder X-Ray diffraction
