# Supplementary material for: Local Induction Heating Capabilities of Zeolites Charged with Metal and Oxide MNPs for Application in HDPE Hydrocracking: A Proof of Concept
Source: Materials (Basel). 2021 Feb 22;14(4):1029. doi: 10.3390/ma14041029 (PMC7926789; doi:10.3390/ma14041029)
Supplement: Supplementary file 1 [file materials-14-01029-s001.zip › materials-1098042-supplementary.pdf]

materials

MDPI

Supplementary Materials

Local Induction Heating Capabilities of Zeolites Charged with Metal and Oxides MNPs for Application in HDPE Hydrocracking: A Proof of Concept

Marta Muñoz *, Irene Morales, Catia S. Costa, Marta Multigner, Patricia de la Presa, Jose M. Alonso, João M. Silva, Maria do Rosário Ribeiro, Belén Torres and Joaquín Rams

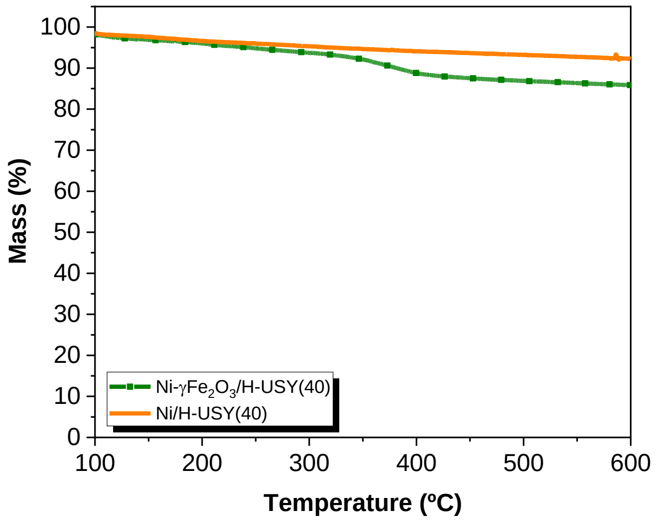

Figure S1. TGA of Ni and Ni- γ-Fe_2O_3 impregnated H-USY(40) zeolites, under hydrogen atmosphere.

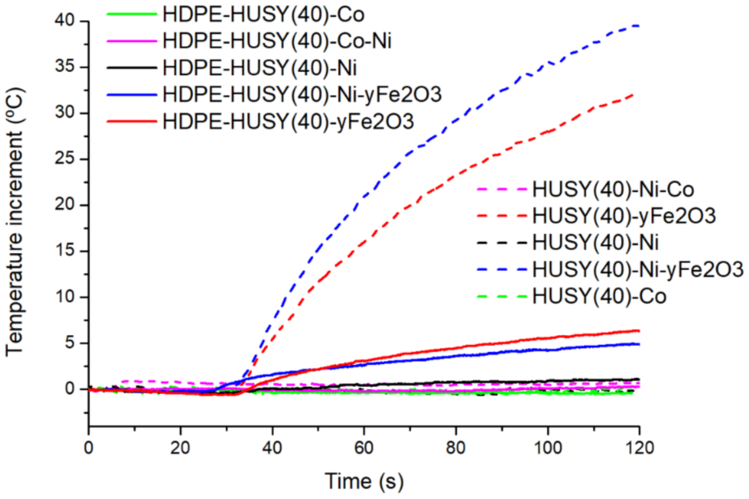

(**a**)

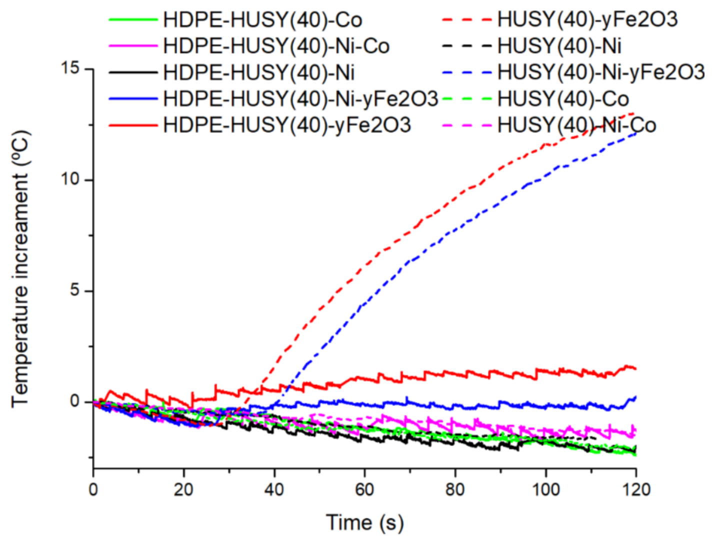

(**b**)

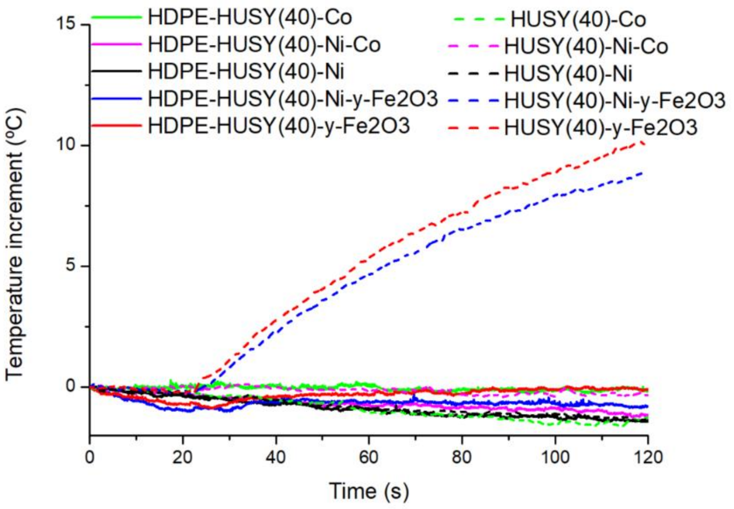

(**c**)

Figure S2. (**a**) Heating curves of all the samples: films with HDPE (continuous lines) and pellets (dashed lines) at 112 kHz. **(b**) Heating curves of all the samples (films and pellets) at 627 kHz. (**c**) Heating curves of all the samples (films and pellets) at 990 kHz.
